# Aromatic Modification of Low Molecular Weight PEI for Enhanced Gene Delivery

**DOI:** 10.3390/polym9080362

**Published:** 2017-08-15

**Authors:** Qing-Ying Yu, Yu-Rong Zhan, Ji Zhang, Chao-Ran Luan, Bing Wang, Xiao-Qi Yu

**Affiliations:** Key Laboratory of Green Chemistry and Technology (Ministry of Education), College of Chemistry, Sichuan University, Chengdu 610064, China; 2016322030015@stu.scu.edu.cn (Q.-Y.Y.); 2014141231224@stu.scu.edu.cn (Y.-R.Z.); 2014222030150@stu.scu.edu.cn (C.-R.L.); wb.chembio@gmail.com (B.W.)

**Keywords:** gene delivery, non-viral gene vector, LMW PEI, aromatic amino acid, structure-activity relationship

## Abstract

Low molecular weight polyethylenimine (1800 Da, also referred to as oligoethylenimines, OEI) was modified with amino acids, including two aromatic amino acids (tryptophan, phenylalanine) and an aliphatic amino acid (leucine). The substitution degree of amino acids could be controlled by adjusting the feeding mole ratio of the reactants. Fluorescence spectroscopy and circular dichroism experiments demonstrated that the indole ring of tryptophan may intercalate into the DNA base pairs and contribute to efficient DNA condensation. In vitro gene expression results revealed that the modified OEIs (OEI-AAs) may provide higher transfection efficiency even than high molecular weight polyethylenimine (25 kDa, PEI), especially the aromatic tryptophan substituted OEI. Moreover, OEI-AAs exhibited excellent serum tolerance, and up to 137 times higher transfection efficiency than PEI 25 kDa that was obtained in the presence of serum. The cytotoxicity of OEI-AAs is much lower than PEI 25 kDa. This study may afford a new method for the development of low molecular weight oligomeric non-viral gene vectors with both high efficiency and biocompatibility.

## 1. Introduction

Nucleic acids are attractive compounds for modulating cell function with high precision [1,2]. Since naked nucleic acids may be easily degraded by nuclease, and the structure of negatively charged phosphates restrict the uptake into cells. Thus, safe and efficient gene delivery vectors are urgently needed [3,4,5,6]. Compared with viral vectors, polycations show lower immunogenicity and a high nucleic acid loading capacity, and they can be easily prepared and their chemical structures may be conveniently modified, exhibiting potential for the construction of ideal non-viral gene delivery systems [7,8,9,10]. Among the “off-the-shelf” polycations, polyethylenimine (PEI) has been regarded as the benchmark for newly designed non-viral polycationic vectors for its high condensation capability toward DNA, strong buffering capacity, and relatively high gene transfection efficiency (TE) [11,12,13]. However, like other polycations for gene delivery, PEI also faces several extracellular and intracellular barriers such as serum stability, cellular uptake, endosomal escape, and nuclear localization [14,15]. The major limitations for its application include the following: firstly, high molecular weight (HMW) PEI (such as 25 kDa) has high TE but considerable toxicity and limited biocompatibility, whereas low molecular weight (LMW) PEI has low toxicity but poor TE [16,17]. It was found that PEI with a molecular weight <2 kDa exhibited almost no in vitro cytotoxicity and low TE even at high concentrations [18,19]. Secondly, PEI has a weak serum tolerance ability, which is necessary for in vivo application [20]. Modification of PEI with some functional groups may overcome specific gene delivery barriers and improve the TE [21,22]. For example, hydrophobic modification of PEI could improve the interaction between the vector–DNA complex (polyplex) and the cell membrane and lead to more efficient cellular uptake [23]. The introduction of negatively charged moieties such as carboxylic acids and phenolic hydroxyls may reduce cytotoxicity while improving the TE [11,24]. Besides, the surface positive charge of PEI could be shielded by a biocompatible polyethyleneglycol (PEG) structure, resulting in an elongated circulation time, reduced cytotoxicity and improved serum tolerance [25,26,27,28]. However, the introduction of negatively charged moieties or PEG may shield the positive charge of PEI and hinder their DNA condensation and cellular uptake ability [29].

Compared to HMW PEI, there are less studies on the decoration of LMW PEI for multiple purposes toward non-viral gene delivery. Most works on LMW PEI were crosslinking with special bridges to form polymers with higher molecular weights [30,31,32,33], and there were much fewer studies on the direct modification of LMW PEI with small molecules [34]. Amino acid modification is an effective strategy to improve the TE of dendrimers [35,36]. Amino acid residues, such as guanidine and imidazole groups, could effectively adjust the physicochemical characteristics of the polycation core [21]. Meanwhile, it was reported that the indole rings may possess strong binding ability toward DNA [37]. We also found that aromatic groups in the polycation structure may improve DNA condensation ability and the subsequent transfection process [16,38]. In this study, we chose three amino acids including tryptophan (W), phenylalanine (F) and leucine (L) to modify LMW PEI (1800 Da, also referred to as oligoethylenimines, OEI) with various substitution degrees (SD) and investigated their effects on the gene delivery process. It was found that the modification could largely enhance the TE together with the serum tolerance, and TE of up to two orders of magnitude higher than PEI 25 kDa could be obtained, indicating a new method for the development of low molecular weight oligomeric non-viral gene vectors with both high efficiency and biocompatibility.

## 2. Experimental Section

### 2.1. Material and Methods

All reagents and chemicals were purchased from commercial providers and used without further purification unless specially noted. Absolute dichloromethane (CH_2_Cl_2_) was distilled after being dried with calcium hydride (CaH_2_). Column chromatography was performed using 200–300 mesh silica gel. All aqueous solutions were prepared from deionized water. Branched polyethylenimine (PEI 25 kDa, *M*_w_ = 2.5 × 10^4^) was supplied by Sigma-Aldrich (St. Louis, MO, USA) and low molecular weight PEI (PEI 1800 Da, *M*_w_ = 1800) was purchased from Aladdin (Shanghai, China). The plasmids used in the study were pEGFP-N1 (Clontech, Palo Alto, CA, USA) coding for Enhanced Green Fluorescent Protein (EGFP) DNA and pGL-3 (Promega, Madison, WI, USA) coding for luciferase DNA. Cy5 was purchased from Mirus Bio, LLC (Madison, WI, USA). The fetal bovine serum (FBS) and Dulbecco’s Modified Eagle Medium (DMEM) were purchased from Invitrogen Corp (Carlsbad, CA, USA). HEK293 human embryonic kidney cell lines and HeLa human cervical cancer cell lines were purchased from the Shanghai Institute of Biochemistry and Cell Biology, Chinese Academy of Sciences (Shanghai, China). The luciferase assay kit and MTS (3-(4,5-dimethylthiazol-2-yl)-5-(3-carboxymethoxy-phenyl)-2-(4-sulfo-phenyl)-2*H* tetrazolium, inner salt) were obtained from Promega (Madison, WI, USA). The ^1^H NMR spectra were measured on a Bruker AM400 NMR spectrometer (Bruker Corporation, Billerica, MA, USA).

The ethidium bromide (EB) displacement assay, particle size and zeta-potential measurement (DLS), cellular uptake of plasmid DNA (flow cytometry) and cytotoxicity were carried out according to our previously reported procedures [16,39,40].

### 2.2. Modification of OEI

*N*-(tert-Butoxycarbonyl)-amino acid (2 mmol), *N*-(3-dimethylaminopropyl)-*N*′-ethylcarbodiimide hydrochloride (EDCI, 2.4 mmol), 1-hydroxybenzotriazole (HOBT, 2.4 mmol), and DIEA (ethyldiisopropylamine, 2.4 mmol) in dry dichloromethane (50 mL) were stirred for 2 h in an ice bath. Then the desired amount of OEI (20%, 30% and 50%, carboxyl/amine in OEI, mol/mol) was dissolved in dry dichloromethane and added to the reaction mixture and stirred for two days at room temperature. After completion of the reaction, diluted HCl solution (0.5 N in water) was added to remove the protecting *t*-butyloxycarbonyl (Boc) group, and the reaction mixture was stirred for another day. The solvent was removed under reduced pressure and the residue with a small amount of water was dialyzed (MWCO 1000 Da) against deionized water for three days. The product was obtained as a white or pale yellow solid after lyophilization. Yield: 16.9–57.5%.

**OEI-W**: ^1^H NMR (400 MHz, D_2_O) δ 7.98–7.16 (m, Ar–*H*), 4.16 (s, –C*H*NH_2_COOH), 3.31–2.73 (m, OEI-*H* and –C*H*_2_CHNH_2_COOH).

**OEI-F**: ^1^H NMR (400 MHz, D_2_O) δ 7.4–7.28 (m, Ph–*H*), 4.12 (s, –C*H*NH_2_COOH), 3.47–2.38 (m, OEI-*H* and –C*H*_2_CHNH_2_COOH).

**OEI-L**: ^1^H NMR (400 MHz, D_2_O) δ 3.50–2.68 (m, OEI–*H* and –CH_2_C*H*NH_2_COOH), 1.65–1.43 (m, (CH_3_)_2_C*H*C*H*_2_–), 0.90 (s, (C*H*_3_)_2_CHCH_2_–).

The substitution degrees (SD), which represents the mole ratio of amino acid to the amine groups in OEI, were calculated from 1H NMR spectra (**OEI-W10** for example, Figure S6A in Supplementary Materials).

### 2.3. Gel Retardation Assay

Polyplexes at different *w/w* ratios (weight ratios of polymer relative to pDNA) were prepared by adding appropriate volumes of the polymer solution to 5 µL of Puc-19 (0.025 mg/mL). The obtained complex solution was then diluted to the total volume of 10 µL. After incubation at 37 °C for 30 min, 2.5 µL of loading buffer was added. Then the mixture was electrophoresed on a 1% (*w*/*v*) agarose gel containing GelRed^TM^ in a Triseacetate (TAE) running buffer at 120 V for 40 min. Then DNA was visualized under an ultraviolet lamp using a BioRad Universal Hood II.

### 2.4. Fluorescence Spectroscopy

Fluorescence spectra were measured at room temperature in air by a HITACHI F-7000 Fluorescence Spectrophotometer (Hitachi High-Tech Science, Schaumburg, IL, USA) and corrected for the system response. Polymers (30 µL, 1 mg/mL) were put into a quartz cuvette containing 2.5 mL Hepes solution. After shaking, the fluorescence intensity of the polymers (F_p_) was measured. Then Calf Thymus (CT) DNA with various *w/w* ratios to polymers was added to the solution and mixed symmetrically, and the measured fluorescence intensity (F) was the result of the interaction between the DNA and the polymers. All the samples were excited at 288 nm and the emission was measured at 378 nm.

### 2.5. Circular Dichroism (CD)

The circular dichroism of DNA was testified by Chirascan (Applied Photophysics, Surrey, UK) with a path length cell of 1 cm at room temperature. CT DNA (1 mg/mL, 100 µL) was added to a Hepes solution (pH 7.4, 2 mL). After the sample was mixed well, the circular dichroism was measured. Then, the solution of polymers (1 mg/mL, 20 or 40 µL) was added each time, and the circular dichroism was measured. The standard scan parameters for all experiments used a wavelength range from 350 to 230 nm. Three scans were made and the average value of them was calculated.

### 2.6. Transmission Electron Microscopy (TEM)

The morphologies of the polyplexes were observed by TEM (Hitachi High-Tech Science, Schaumburg, IL, USA) with an acceleration voltage of 100 kV. A total of 4 µg of pUC-19 was added to the appropriate volume of the polymer solution (weight ratio of polymer relative to pDNA, *w/w* = 16:1 for **OEI-W10**&**OEI-L10** and *w/w* = 24:1 for **OEI-F10**&**OEI**), and incubated at 37 °C for 0.5 h. Then the polyplex solution was diluted to the total volume of 200 µL. A drop of DNA/polymer polyplex suspension was placed onto the copper grid. After a few minutes, the excess solution was blotted away with filter paper. Then, a drop of 0.5% (*w*/*v*) phosphotungstic acid was placed on the above grid. The grid was dried at room temperature at atmospheric pressure for several minutes before observation.

### 2.7. Gene Transfection Efficiency Assay In Vitro

For the expression of the EGFP gene Hela and HEK293 cells were seeded at a density of 1 × 10^5^ cells per well in a 24-well plate in DMEM containing 10% FBS and grown to reach 70–80% confluence prior to transfection. Then, the medium of each well was exchanged for fresh, serum-free medium. Subsequently, the cells were treated with polyplexes (containing 0.8 µg of pEGFP) at different (*w*/*w*) weight ratios and PEI/DNA at a weight ratio of 1.4 (N/P = 10) for 4 h at 37 °C. The medium was then completely refreshed with the completed culture media. 24 h after transfection, cells were observed with an inverted fluorescence microscope (Nikon Eclipse TS 100, Nikon Corporation, Tokyo, Japan) equipped with a cold Nikon camera. Digital image recording and image analysis were performed with the NIS Elements Advanced Research software (version 4.0).

For the luciferase assays, cells were transfected by polyplexes containing pGL-3. For a typical assay in a 24-well plate, 24 h post transfection as described above, cells were washed with warm PBS and lysed with 100 µL 1 × lysis reporter buffer (Promega, Fitchburg, MA, USA). The luciferase activity was measured by microplate reader (Model 550, BioRad, Hercules, CA, USA). The protein concentration in the cell lysate supernatant was estimated in each case with a Lowry protein assay kit (PIERCE, Rockford, IL, USA). Gene transfection efficiency was expressed as the relative fluorescence intensity per mg of protein (RLU/mg protein). All the experiments were done in triplicate.

## 3. Results and Discussion

### 3.1. Synthesis and Characterization of the Modified OEIs (OEI-AAs)

**Scheme 1:**
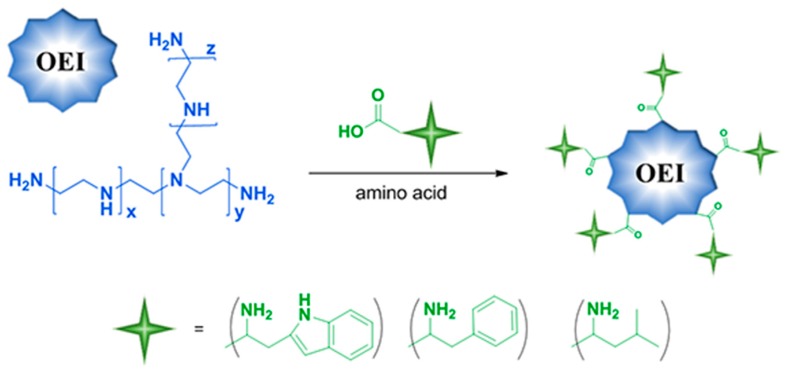
Formation of OEI-AA from OEI.

As shown in Scheme 1, tryptophan (W), phenylalanine (F) and leucine (L), which contain aromatic indole, aromatic phenyl and aliphatic *iso*-butyl group, respectively, were chosen to modify OEI (1800 Da) with various substitution degrees (SD). The primary amine groups of the amino acids were previously protected by the Boc group. The Boc-amino acids were then coupled with OEI through the formation of amide groups. The SD, which may be calculated from the specific peak integrals of the ^1^H NMR spectra, was controlled by adjusting the feeding mole ratio of amino acids toward the amine groups in OEI (*M*_W_ = 43 g/mol for each ethylenimine unit). Generally, for the steric effect and the reactivity diversity of 1°/2°/3° amines in OEI, the obtained SDs were lower than the relative feeding ratio (Table 1). It is worth noting that the number in the product name refers to the actual (calculated) SD, e.g., **OEI-W10** means that the SD of tryptophan is about 10%. Due to the various structures of different amino acids, the same feeding ratio would not lead to OEI-AA with similar SDs. High SD was hard to achieve for the steric issue, while very low SD could not exhibit the role of amino acids. Thus, we chose products with SDs in the range of around 10–20%, which are listed in Table 1.

### 3.2. Interaction with DNA and Formation of Polyplex

**Figure 1:**
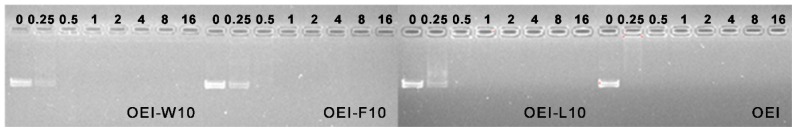
DNA condensation by OEI-AAs at various weight ratios evaluated by an agarose gel retardation assay.

The primary requirement for polymeric gene vectors is the ability to bind and condense plasmid DNA. Agarose gel retardation assay was first used to estimate the DNA-binding ability of these polycations. As shown in Figure 1, the studied OEI-AAs could completely retard DNA migration at a small polymer/DNA weight ratio (*w*/*w*) of 0.5. On the other hand, this *w*/*w* was slightly larger than the cases using OEI or PEI 25 kDa (Figure S1), suggesting that the surface modification of OEI might have negative steric effect on the interaction with DNA. However, subsequent experiments revealed that the transfection efficiency could be largely improved by the modification, indicating that excessively strong DNA binding might hinder the DNA release and gene transfection. The ethidium bromide (EB) exclusion experiment gave consistent results (Figure S2). All the tested materials could efficiently displace EB that previously intercalated into DNA base pairs, leading to fluorescence quenching. Unmodified OEI and PEI could quench the fluorescence of EB more quickly than the modified forms, but the difference was small. **OEI-W** and **OEI-F** had slightly better binding ability than **OEI-L**, indicating that the aromatic rings might facilitate their interaction with DNA. To examine the special property of aromatic rings toward DNA binding, we also studied the fluorescence change of **OEI-W** before and after its interaction with DNA. Results show that the fluorescence intensity of **OEI-W10** at 354 nm (the characteristic peak corresponding to the tryptophan residues) gradually lowered with the increase in the amount of DNA (decrease of *w*/*w*, Figure S3), suggesting that the aromatic indole rings of tryptophan might intercalate into the DNA base pairs, leading to distinct fluorescence change [37]. On the contrary, a similar phenomenon was not observed for **OEI-F10**, which has aromatic phenyl rings in the structure. Thus, it was believed that the indole ring may play special role in the interaction with DNA. Further, heparin was used to study the release of DNA from the polyplexes. It was found that DNA could be gradually released with the increasing amount of heparin (Figure S4). Similar to the above results, since OEI has a higher DNA-binding ability than the modified forms, more heparin was needed to release the DNA from the polyplex formed from OEI.

**Figure 2:**
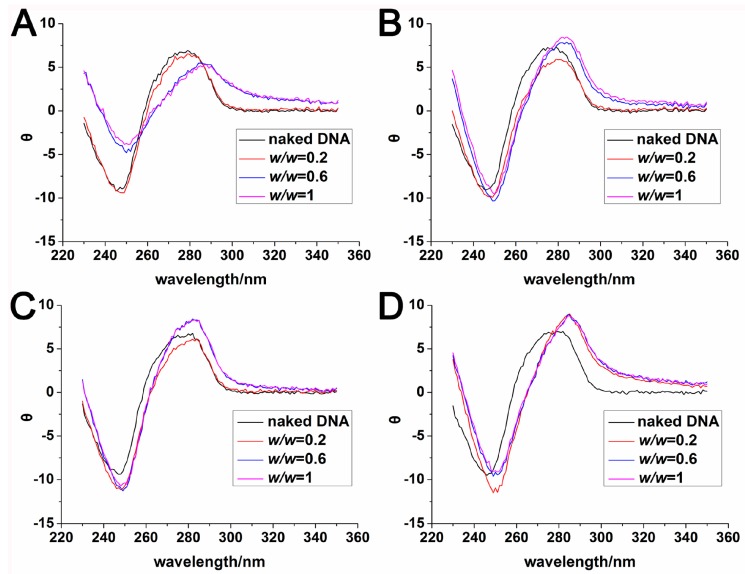
CD spectra of DNA/polycations ((**A**) **OEI-W10**; (**B**) **OEI-F10**; (**C**) **OEI-L10**; (**D**) **OEI**) with different *w/w* ratios.

Free B-type DNA shows a typical circular dichroism (CD) spectrum, and the addition of polycations would make special changes on the CD spectra. As shown in Figure 2, with the addition of **OEI-W10**, the molar ellipticities of DNA at 245 nm and 279 nm were distinctly suppressed from a *w*/*w* of 0.6 together with a certain quantity of red shift. We speculate that the signal change might be ascribed to the intercalation of indole rings into the DNA base pairs [16,41]. On the other hand, other polycations just led to a slight red shift but no obvious suppression of molar ellipticity. These results also indicated the unique role of indole or tryptophan in DNA binding.

**Figure 3:**
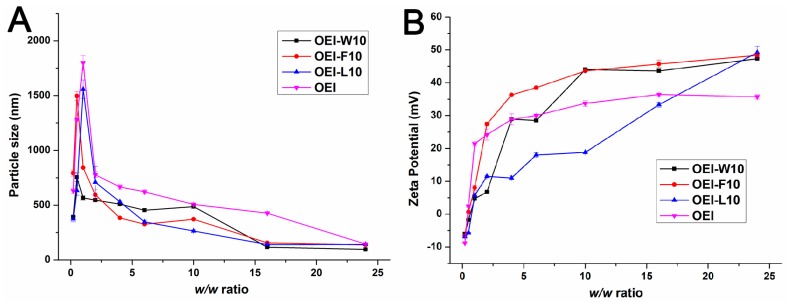
Particle size (**A**) and zeta potential (**B**) of polyplexes obtained at different weight ratios by DLS. Data represent mean ± SD (*n* = 3).

**Figure 4:**
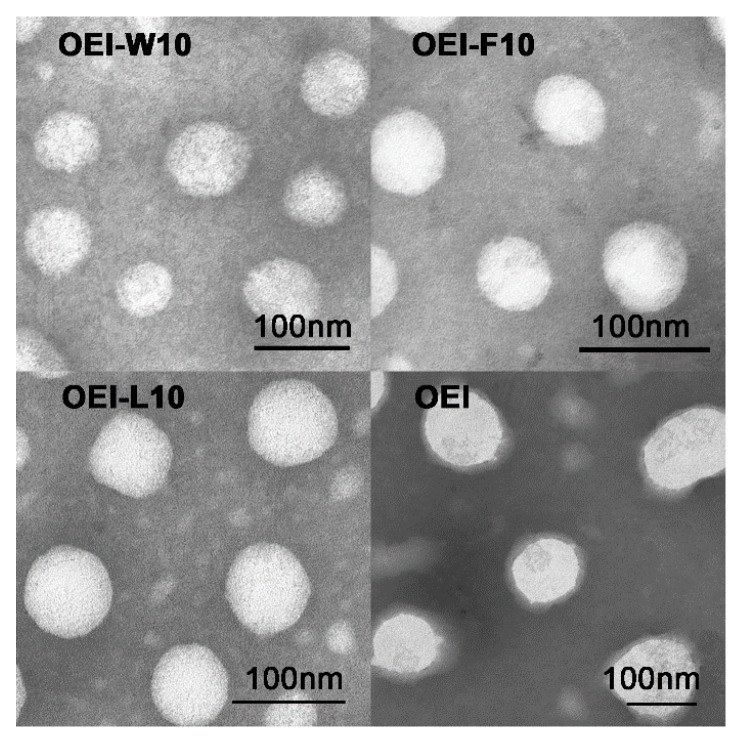
TEM image of polyplexes at certain weight ratio (*w*/*w* = 16:1 for OEI-W10&OEI-L10 and *w/w* = 24:1 for OEI-F10&OEI) in deionized water.

The physical properties such as particle size and zeta potential of the formed polyplexes were subsequently studied by the dynamic light scattering (DLS) method. The results in Figure 3A show that the particle size became stable from the *w*/*w* of six for all polycationic materials, and the values were about 100~500 nm, which is appropriate for cellular uptake [42]. Although the OEI-AAs showed slightly weaker DNA-binding ability than OEI, the particles formed from OEI-AAs were smaller, indicating their better DNA condensation ability. Plasmid DNA is negatively charged in neutral conditions. With the addition of OEI-AAs, the zeta potential of the polyplexes turned positive at a relatively low *w*/*w*, <1, and finally reached a plateau of +47~50 mV (Figure 3B). Such a positive charge might increase their interaction with the negatively charged cell membrane and promote cellular uptake. Moreover, the morphology of the polyplex was directly observed by TEM. The images in Figure 4 show that in deionized water, all materials could condense DNA into uniform regular spherical nanoparticles with a diameter of 80 ± 20 nm. The discrepancy between the sizes measured by DLS and TEM could be ascribed to the different environments of the testing sample—DLS determined the hydrodynamic diameter of the particles in water, while TEM showed the morphology of the particles in their dehydrated state [43].

### 3.3. In Vitro Gene Transfection

**Figure 5:**
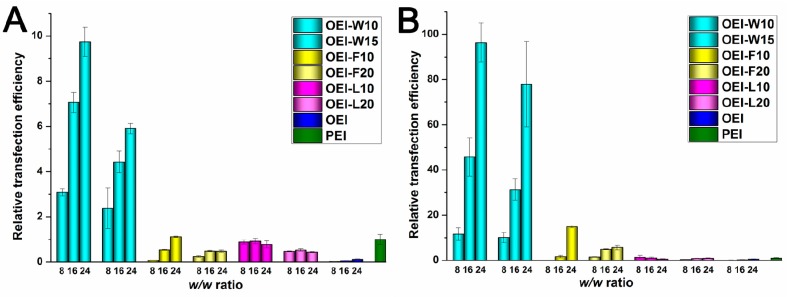
Luciferase gene expression transfected by polyplexes at different weight ratios without (**A**) and with (**B**) the presence of 10% serum in HEK293 cells by normalizing the TE of PEI (*w*/*w* = 1.4) as 1. Data represent mean ± SD (*n* = 3).

To investigate the gene transfection efficiency of OEI-AAs, the luciferase reporter gene pGL-3 plasmid was first used to quantitatively assess their in vitro TE in HEK293 cells. Figure 5 shows the transfection results, which were described as relative TE by normalizing the TE of PEI (*w/w* = 1.4, N/P = 10) as 1. It is well known that low molecular weight PEI (e.g., OEI) is less toxic than its higher molecular weight counterpart such as PEI 25 kDa, but its TE is lower. This was demonstrated by the experiments, in which OEI indeed showed much lower TE. On the contrary, to our delight, the modification of OEI with amino acids was able to greatly improve the TE, which was even higher than that of PEI 25 kDa. OEI modified by tryptophan exhibited the best transfection results, and the TE reached levels up to ten times higher than PEI (**OEI-W10**). The modification with phenylalanine or leucine was also able to enhance the TE of OEI, but to a lesser extent. The optimal TE of **OEI-F** and **OEI-L** were similar to PEI. On the other hand, results also show that OEI-AAs with an SD of 10% are more suitable for transfection. Further, the transfection experiments were also carried out in serum circumstances to estimate the serum tolerance of the materials (Figure 5B). With the presence of 10% serum, the OEIs modified by the amino acids containing aromatic rings were able to give much higher relative TE than those without serum, especially for **OEI-W10**, which gave 96 times higher TE than PEI. We speculate that the excellent serum tolerance might come from their good DNA condensation ability, making the polyplex more stable.

**Figure 6:**
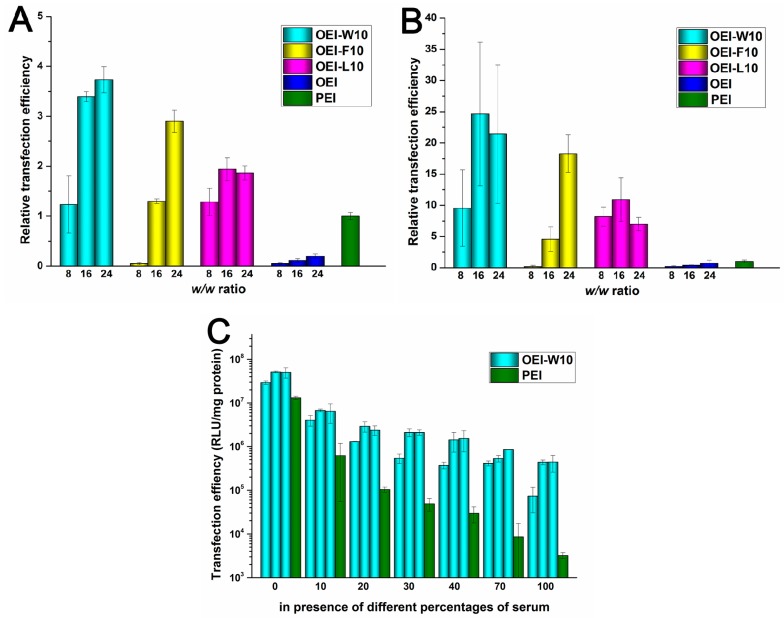
Luciferase gene expression transfected by polyplexes at different weight ratios ((**A**,**B**) Relative TE by normalizing the TE of PEI (*w*/*w* = 1.4) as 1; (**C**) absolute TE) in HeLa cells. Data represent mean ± SD (*n* = 3). (**A**) Without serum; (**B**) With the presence of 10% serum; (**C**) Effect of serum concentration on the TE (weight ratio for **OEI-W10**: 8, 16 and 24).

The OEI-AAs with 10% SD were also applied to the transfection toward tumor cells (HeLa). As shown in Figure 6A, the results were similar to those obtained in HEK293 cells. **OEI-W10** showed the highest TE, and all of the OEI-AAs had higher TE than PEI. The green fluorescent protein (GFP) transfection assay (Figure S5) visually revealed that OEI induced much less green fluorescence than PEI, but the modified form could improve the TE, especially the tryptophan-substituted **OEI-W**. With the presence of serum, the relative TE of OEI-AAs increased significantly (Figure 6B), and **OEI-W10** showed 25 times higher levels of TE than PEI, further indicating its excellent serum tolerance. **OEI-F10** and **OEI-L10** could also exhibit much better transfection than PEI in serum circumstances. Moreover, the effect of serum concentrations on the TE was also studied (Figure 6C). It was confirmed that the TE of **OEI-W10** was less negatively affected by serum than that of PEI, and the relative TE (PEI as standard) continuously increased along with the rise of serum concentration.

**Figure 7:**
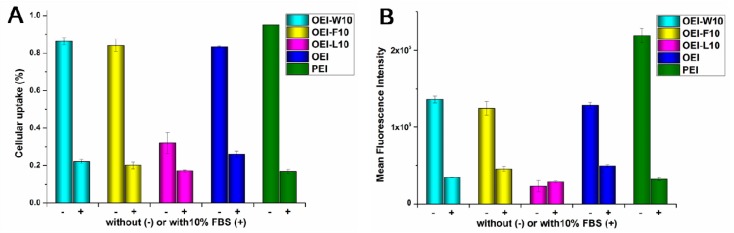
Cellular uptake of polyplexes at the optimal transfection *w*/*w* ratio in HeLa cells quantified by flow cytometry analysis. (**A**) Percentage of uptake cells; (**B**) mean fluorescence intensity of transferred DNA. Data represent mean ± SD (*n* = 3).

To figure out the positive effect of amino acid modification on both TE and serum tolerance, the cellular uptake of the polyplexes was subsequently studied. Flow cytometry was used to measure the HeLa cell internalization by using Cy5-labelled DNA via the fluorescence activated cell sorting (FACS) technique. After 4 h incubation with the polyplexes, the percentage of cells positive for Cy5-labelled pDNA and mean fluorescence intensity were calculated and shown in Figure 7. To our surprise, in the absence of serum, both the uptake cell percentage and fluorescence intensity induced by OEI and OEI-AAs were lower than those by PEI. Thus the higher TE of OEI-AAs might come from more efficient intracellular delivery [44]. We postulate that polyplexes formed from higher molecular weight PEI would suffer more difficult DNA release, leading to lower TE [31]. With the addition of serum, the uptake cell percentage and fluorescence intensity induced by PEI were severely inhibited, and the results were at the same level as those of OEI-AAs. In other words, the decrease of cellular uptake of OEI-AA/DNA polyplexes was less than that of PEI/DNA, suggesting that OEI-AAs have better serum tolerance ability.

### 3.4. In Vitro Cytotoxicity

**Figure 8:**
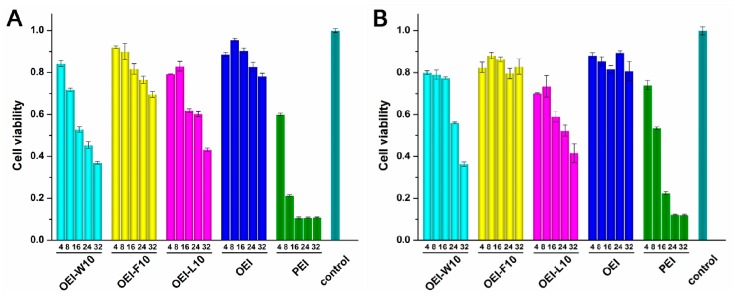
Cytotoxicity of polyplexes at various weight ratios in HEK293 (**A**) and HeLa cells (**B**).

The cell viability of the polyplexes prepared at various weight ratios was evaluated by comparison with that of PEI/DNA in HEK293 and HeLa cells by using an MTS assay. As shown in Figure 8, the cell viability of the polyplexes formed from OEI-AAs is indeed a bit lower than OEI, indicating that modification with amino acids would lead to slight increase of cytotoxicity. This might be due to the increase of the molecular weight via the modification [45,46]. However, the cytotoxicities under optimal transfection *w*/*w* ratios (16~24) were acceptable. On the other hand, the cytotoxicity of OEI-AAs was much lower than PEI, especially at higher weight ratios. Compared to PEI 25 kDa, the much higher TE together with lower cytotoxicity made the OEI-AAs good candidates for further applications.

## 4. Conclusions

In summary, PEI with a relatively low molecular weight (1800 Da, also described as OEI) was modified with three amino acids to study the gene delivery capability of the target materials. The substitution degree of the amino acids could be calculated from the specific peak integrals in the ^1^H NMR spectra. It was found that the modification would slightly reduce the DNA binding ability, but the OEI-AAs could condense DNA into more compact nanoparticles. CD results indicated that the indole ring of tryptophan might intercalate into the DNA base pairs, resulting in more efficient condensation. In vitro transfection experiments revealed that the modification of OEI, especially with aromatic amino acids, could largely improve the TE, which was even much higher than PEI 25 kDa. Moreover, these OEI-AAs exhibited excellent serum tolerance, which PEI lacks. Up to 137 times higher TE than can be found in PEI was obtained by using **OEI-W10** as vector. These materials with high TE together with acceptable cytotoxicity may be promising gene vectors for future applications.

## Figures and Tables

**Table 1:** The products obtained by the modification of OEI and their SD calculated from ^1^H NMR spectra.

Amino Acid	Tryptophan (W)		Phenylalanine (F)		Leucine (L)	
Product	OEI-W10	OEI-W15	OEI-F10	OEI-F20	OEI-L10	OEI-L20
Feeding ratio (%)	20.0	30.0	30.0	50.0	30.0	50.0
Calculated SD (%)	10.0	14.2	9.0	18.6	9.6	22.2

## Supplementary Materials

The following are available online at www.mdpi.com/2073-4360/9/8/362/s1, Figure S1: DNA condensation by polycations at various weight ratios evaluated by agarose gel retardation assay. Figure S2: Fluorescence quenching assay of EB/DNA by addition of polycations. Figure S3: Fluorescence spectra of **OEI-W10** upon the addition of CT DNA in 2.5 mL Hepes solution (excited at 288 nm) at room temperature. Figure S4: Release of DNA from polyplxes with the addition of heparin at various heparin/DNA weight ratios. *w*/*w* = 0, 0.25, 0.5, 1, 2, 4, 8, 16, 32, 64; polymer/DNA: *w*/*w* = 1. Figure S5: Fluorescence microscopy image of pEGFP-transfected HEK293 and HeLa cells in the absence serum. Figure S6: (**A**) ^1^H NMR spectra (400 MHz, D_2_O) of **OEI-W10**; (**B**) ^1^H NMR spectra (400 MHz, D_2_O) of **OEI-W15**; (**C**) ^1^H NMR spectra (400 MHz, D_2_O) of **OEI-F10**; (**D**) ^1^H NMR spectra (400 MHz, D_2_O) of **OEI-F20**; (**E**) ^1^H NMR spectra (400 MHz, D_2_O) of **OEI-L10**; (**F**) ^1^H NMR spectra (400 MHz, D_2_O) of **OEI-L20**. The characteristic multiplet of δ 7.58–7.16 represents the 5H on indole ring of tryptophan (W), while the broad multiplet represents the C-H of PEI (4H for each ethylenimine unit) and 2H on the -CHCH_2_-on W. The SD can be calculated from the ratio between these peak areas. e.g., in this case, for **OEI-W1****0**, SD = n(W)/n(ethylenimine units on OEI) = (5/5)/[(41.87 − 2)/4] ≈ 0.10, i.e., 10%.

## References

[1] Lachelt U., Wagner E. (2015). Nucleic acid therapeutics using polyplexes: A journey of 50 years (and beyond). Chem. Rev..

[2] Chiper M., Tounsi N., Kole R., Kichler A., Zuber G. (2016). Self-aggregating 1.8 kDa polyethylenimines with dissolution switch at endosomal acidic pH are delivery carriers for plasmid DNA, mrna, sirna and exon-skipping oligonucleotides. J. Control. Release.

[3] Zaghloul E.M., Viola J.R., Zuber G., Smith C.I.E., Lundin K.E. (2010). Formulation and delivery of splice-correction antisense oligonucleotides by amino acid modified polyethylenimine. Mol. Pharm..

[4] Yu Q.Y., Liu Y.H., Huang Z., Zhang J., Luan C.R., Zhang Q.F., Yu X.Q. (2016). Bio-reducible polycations from ring-opening polymerization as potential gene delivery vehicles. Org. Biomol. Chem..

[5] Niven R., Pearlman R., Wedeking T., Mackeigan J., Noker P., Simpson-Herren L., Smith J.G. (1998). Biodistribution of radiolabeled lipid–DNA complexes and DNA in mice. J. Pharm. Sci..

[6] Kim T.H., Jiang H.L., Jere D., Park I.K., Cho M.H., Nah J.W., Choi Y.J., Akaike T., Cho C.S. (2007). Chemical modification of chitosan as a gene carrier in vitro and in vivo. Prog. Polym. Sci..

[7] Chen C.K., Jones C.H., Mistriotis P., Yu Y., Ma X.N., Ravikrishnan A., Jiang M., Andreadis S.T., Pfeifer B.A., Cheng C. (2013). Poly(ethylene glycol)-*block*-cationic polylactide nanocomplexes of differing charge density for gene delivery. Biomaterials.

[8] Yan X.H., Blacklock J., Li J.B., Möhwald H. (2012). One-pot synthesis of polypeptide–gold nanoconjugates for in vitro gene transfection. ACS Nano.

[9] Mintzer M.A., Simanek E.E. (2009). Nonviral vectors for gene delivery. Chem. Rev..

[10] Yin H., Kanasty R.L., Eltoukhy A.A., Vegas A.J., Dorkin J.R., Anderson D.G. (2014). Non-viral vectors for gene-based therapy. Nat. Rev. Genet..

[11] He Y.Y., Cheng G., Xie L., Nie Y., He B., Gu Z.W. (2013). Polyethyleneimine/DNA polyplexes with reduction-sensitive hyaluronic acid derivatives shielding for targeted gene delivery. Biomaterials.

[12] Wang F., Deng L.F., Hu J.J., Cheng Y.Y. (2016). Being two is better than being one: A facile strategy to fabricate multicomponent nanoparticles for efficient gene delivery. Bioconj. Chem..

[13] Lungwitz U., Breunig M., Blunk T., Göpferich A. (2005). Polyethylenimine-based non-viral gene delivery systems. Eur. J. Pharm. Biopharm..

[14] Kannan R.M., Nance E., Kannan S., Tomalia D.A. (2014). Emerging concepts in dendrimer-based nanomedicine: From design principles to clinical applications. J. Intern. Med..

[15] Tian W.D., Ma Y.Q. (2013). Theoretical and computational studies of dendrimers as delivery vectors. Chem. Soc. Rev..

[16] Luan C.R., Liu Y.H., Zhang J., Yu Q.Y., Huang Z., Wang B., Yu X.Q. (2016). Low molecular weight oligomers with aromatic backbone as efficient nonviral gene vectors. ACS Appl. Mater. Interfaces.

[17] Zhang Q.F., Luan C.R., Yin D.X., Zhang J., Liu Y.H., Peng Q., Xu Y., Yu X.Q. (2015). Amino acid-modified polyethylenimines with enhanced gene delivery efficiency and biocompatibility. Polymers.

[18] Thomas M., Ge Q., Lu J.J., Chen J.Z., Klibanov A. (2005). Cross-linked small polyethylenimines: While still nontoxic, deliver DNA efficiently to mammalian cells in vitro and in vivo. Pharm. Res..

[19] Godbey W.T., Wu K.K., Mikos A.G. (1999). Size matters: Molecular weight affects the efficiency of poly(ethylenimine) as a gene delivery vehicle. J. Biomed. Mater. Res..

[20] Ewe A., Przybylski S., Burkhardt J., Janke A., Appelhans D., Aigner A. (2016). A novel tyrosine-modified low molecular weight polyethylenimine (P10Y) for efficient sirna delivery in vitro and in vivo. J. Control. Release.

[21] Yang J.P., Zhang Q., Chang H., Cheng Y.Y. (2015). Surface-engineered dendrimers in gene delivery. Chem. Rev..

[22] Wang M.M., Liu H., Li L., Cheng Y.M. (2014). A fluorinated dendrimer achieves excellent gene transfection efficacy at extremely low nitrogen to phosphorus ratios. Nat. Commun..

[23] Fu C.L., Lin L., Shi H.L., Zheng D.X., Wang W., Gao S.Q., Zhao Y.F., Tian H.Y., Zhu X.J., Chen X.S. (2012). Hydrophobic poly(amino acid) modified PEI mediated delivery of rev-casp-3 for cancer therapy. Biomaterials.

[24] He Y.Y., Nie Y., Cheng G., Xie L., Shen Y.Q., Gu Z.W. (2014). Viral mimicking ternary polyplexes: A reduction-controlled hierarchical unpacking vector for gene delivery. Adv. Mater..

[25] Hwang A.A., Lee B.Y., Clemens D.L., Dillon B.J., Zink J.I., Horwitz M.A. (2015). PH-responsive isoniazid-loaded nanoparticles markedly improve tuberculosis treatment in mice. Small.

[26] Golani-Armon A., Golan M., Shamay Y., Raviv L., David A. (2015). DC3-decorated polyplexes for targeted gene delivery into dendritic cells. Bioconj. Chem..

[27] Fitzsimmons R.E.B., Uludağ H. (2012). Specific effects of PEGylation on gene delivery efficacy of polyethylenimine: Interplay between PEG substitution and N/P ratio. Acta Biomater..

[28] Kumagai M., Shimoda S., Wakabayashi R., Kunisawa Y., Ishii T., Osada K., Itaka K., Nishiyama N., Kataoka K., Nakano K. (2012). Effective transgene expression without toxicity by intraperitoneal administration of PEG-detachable polyplex micelles in mice with peritoneal dissemination. J. Control. Release.

[29] Petersen H., Merdan T., Kunath K., Fischer D., Kissel T. (2002). Poly(ethylenimine-*co*-l-lactamide-*co*-succinamide):  A biodegradable polyethylenimine derivative with an advantageous ph-dependent hydrolytic degradation for gene delivery. Bioconj. Chem..

[30] Xun M.M., Zhang X.C., Zhang J., Jiang Q.Q., Yi W.J., Zhu W., Yu X.Q. (2013). Low molecular weight PEI-based biodegradable lipopolymers as gene delivery vectors. Org. Biomol. Chem..

[31] Xun M.M., Xiao Y.P., Zhang J., Liu Y.H., Peng Q., Guo Q., Wu W.X., Xu Y., Yu X.Q. (2015). Low molecular weight PEI-based polycationic gene vectors via michael addition polymerization with improved serum-tolerance. Polymer.

[32] Zhang G.Y., Liu J., Yang Q.Z., Zhuo R.X., Jiang X.L. (2012). Disulfide-containing brushed polyethylenimine derivative synthesized by click chemistry for nonviral gene delivery. Bioconj. Chem..

[33] Fang G., Zeng F., Yu C.M., Wu S. (2014). Low molecular weight peis modified by hydrazone-based crosslinker and betaine as improved gene carriers. Colloids Surf. B.

[34] Teo P.Y., Yang C., Hedrick J.L., Engler A.C., Coady D.J., Ghaem-Maghami S., George A.J.T., Yang Y.Y. (2013). Hydrophobic modification of low molecular weight polyethylenimine for improved gene transfection. Biomaterials.

[35] Liu C., Liu X.X., Rocchi P., Qu F.Q., Iovanna J.L., Peng L. (2014). Arginine-terminated generation 4 PAMAM dendrimer as an effective nanovector for functional sirna delivery in vitro and in vivo. Bioconj. Chem..

[36] Choi J.S., Nam K., Park J.Y., Kim J.B., Lee J.K., Park J.S. (2004). Enhanced transfection efficiency of pamam dendrimer by surface modification with l-arginine. J. Control. Release.

[37] Yang K., Chang Y.C., Wen J., Lu Y.C., Pei Y.X., Cao S.P., Wang F., Pei Z.C. (2016). Supramolecular vesicles based on complex of trp-modified pillar[5]arene and galactose derivative for synergistic and targeted drug delivery. Chem. Mater..

[38] Yi W.J., Yu X.C., Wang B., Zhang J., Yu Q.Y., Zhou X.D., Yu X.Q. (2014). Tacn-based oligomers with aromatic backbones for efficient nucleic acid delivery. Chem. Commun..

[39] Zhang Q.F., Yi W.J., Wang B., Zhang J., Ren L., Chen Q.M., Guo L., Yu X.Q. (2013). Linear polycations by ring-opening polymerization as non-viral gene delivery vectors. Biomaterials.

[40] Zhang Q.F., Wang B., Yin D.X., Zhang J., Wu W.X., Yu Q.Y., Yu X.Q. (2014). Linear TACN-based cationic polymers as non-viral gene vectors. RSC Adv..

[41] Li C., Zhao F.F., Huang Y.N., Liu X.Y., Liu Y., Qiao R.Z., Zhao Y.F. (2012). Metal-free DNA linearized nuclease based on PASP–polyamine conjugates. Bioconj. Chem..

[42] Zhou Y.F., Yang B., Ren X.Y., Liu Z.Z., Deng Z., Chen L.M., Deng Y.B., Zhang L.M., Yang L.Q. (2012). Hyperbranched cationic amylopectin derivatives for gene delivery. Biomaterials.

[43] Wang H.F., Luo X.H., Liu C.W., Feng J., Zhang X.Z., Zhuo R.X. (2012). A smart micellar system with an amine-containing polycarbonate shell. Acta Biomater..

[44] Gabrielson N.P., Pack D.W. (2006). Acetylation of polyethylenimine enhances gene delivery via weakened polymer/DNA interactions. Biomacromolecules.

[45] Parhamifar L., Larsen A.K., Hunter A.C., Andresen T.L., Moghimi S.M. (2010). Polycation cytotoxicity: A delicate matter for nucleic acid therapy-focus on polyethylenimine. Soft Matter.

[46] Tripathi S.K., Gupta S., Gupta K.C., Kumar P. (2013). Efficient DNA and sirna delivery with biodegradable cationic hyaluronic acid conjugates. RSC Adv..

