# Supplementary material for: Aromatic Modification of Low Molecular Weight PEI for Enhanced Gene Delivery
Source: Polymers (Basel). 2017 Aug 15;9(8):362. doi: 10.3390/polym9080362 (PMC6418655; doi:10.3390/polym9080362)
Supplement: Supplementary file 1 [file polymers-09-00362-s001.pdf]

Supplementary Materials: Aromatic Modification of Low Molecular Weight PEI for Enhanced Gene Delivery

Qing-Ying Yu, Yu-Rong Zhan, Ji Zhang, Chao-Ran Luan, Bing Wang, and Xiao-Qi Yu

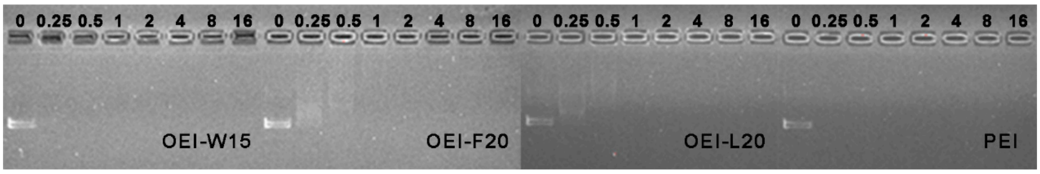

Figure S1. DNA condensation by polycations at various weight ratios evaluated by agarose gel retardation assay.

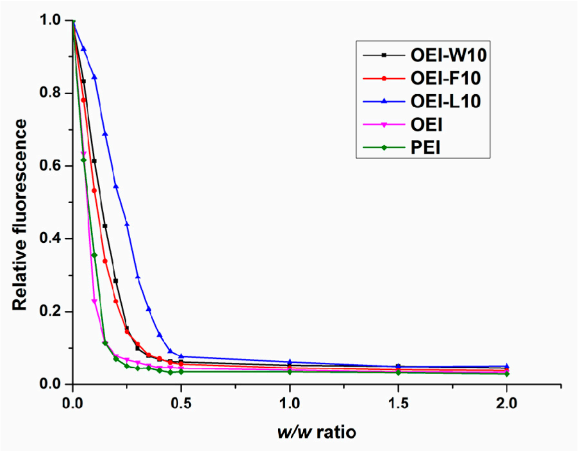

Figure S2. Fluorescence quenching assay of EB/DNA by addition of polycations.

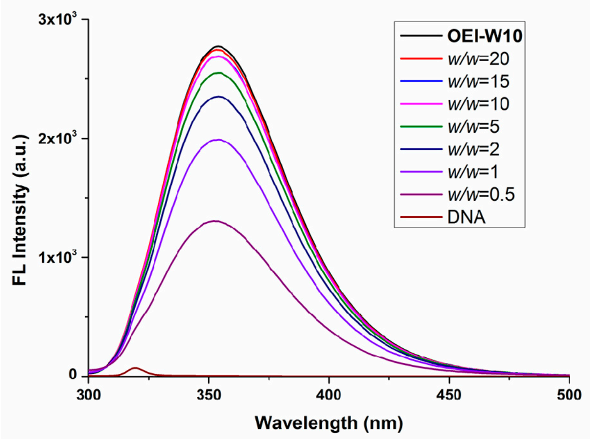

Figure S3. Fluorescence spectra of **OEI-W10** upon the addition of CT DNA in 2.5 mL Hepes solution (excited at 288 nm) at room temperature.

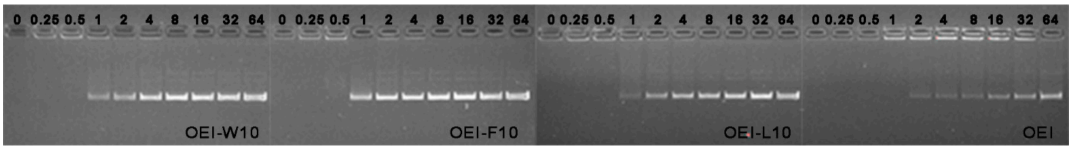

Figure S4. Release of DNA from polyplxes with the addition of heparin at various heparin/DNA weight ratios. *w*/*w* = 0, 0.25, 0.5, 1, 2, 4, 8, 16, 32, 64; polymer/DNA: *w*/*w* = 1

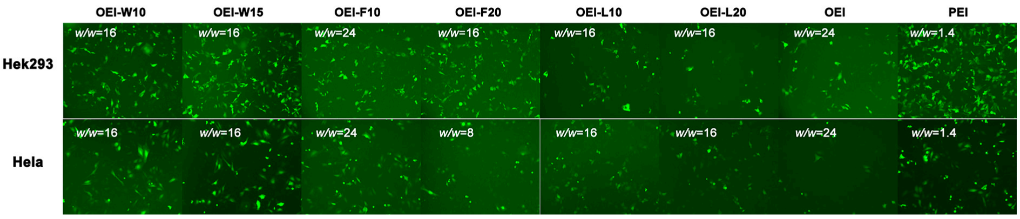

Figure S5. Fluorescence microscopy image of pEGFP-transfected HEK293 and HeLa cells in the absence serum.

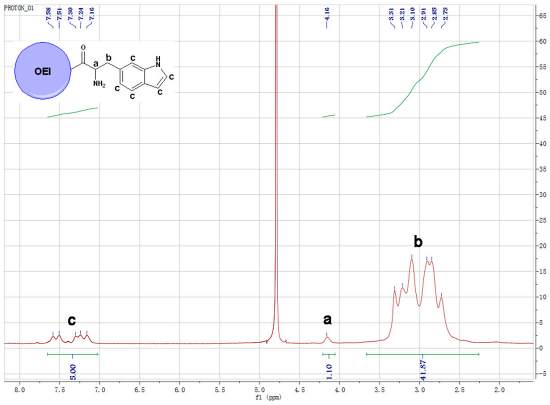

(**A**)

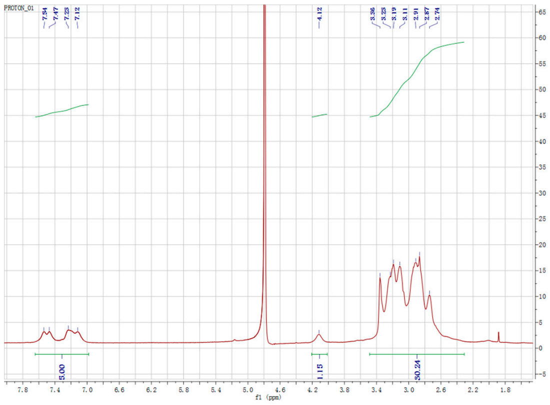

(**B**)

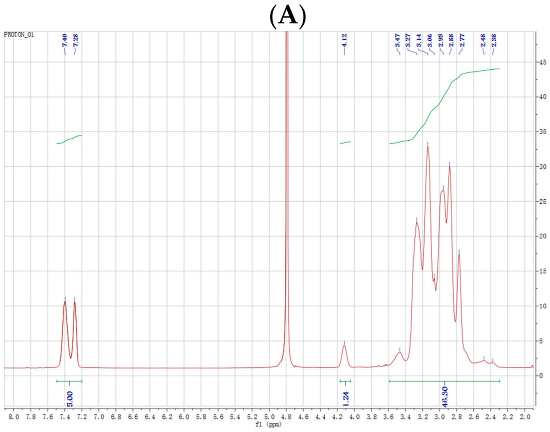

(**C**)

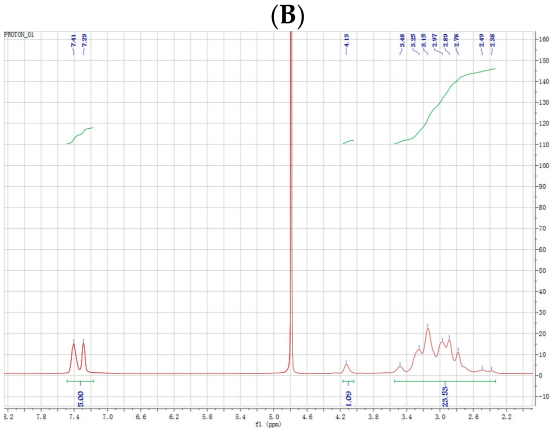

(**D**)

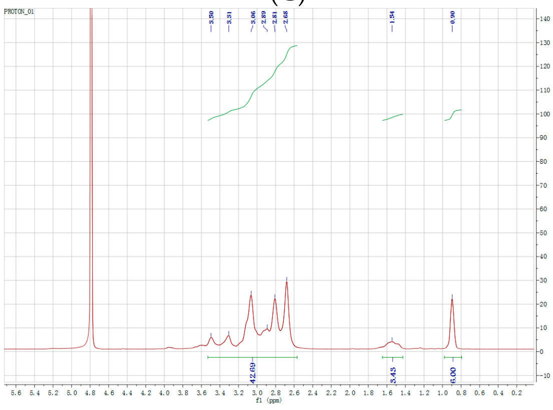

(**E**)

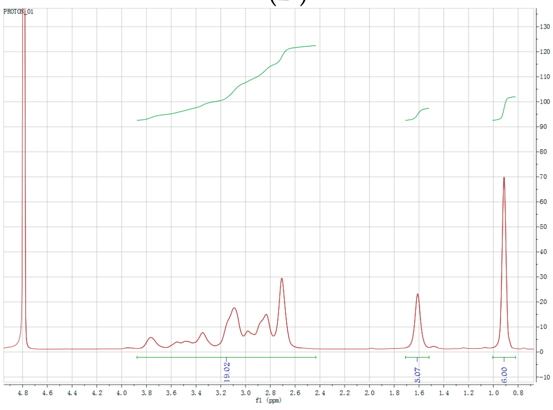

(**F**)

Figure S6. (**A**)^{1}H NMR spectra (400 MHz, D_2O) of **OEI-W10**; (**B**) ^{1}H NMR spectra (400 MHz, D_2O) of **OEI-W15**; (**C**) ^{1}H NMR spectra (400 MHz, D_2O) of **OEI-F10**; (**D**) ^{1}H NMR spectra (400 MHz, D_2O) of **OEI-F20**; (**E**) ^{1}H NMR spectra (400 MHz, D_2O) of **OEI-L10**; (**F**) ^{1}H NMR spectra (400 MHz, D_2O) of **OEI-L20.** The characteristic multiplet of δ 7.58-7.16 represents the 5H on indole ring of tryptophan (W), while the broad multiplet represents the C-H of PEI (4H for each ethylenimine unit) and 2H on the -$CHCH_2$- on W. The SD can be calculated from the ratio between these peak areas. e.g.: In this case, for **OEI-W10**, SD = n (W)/n(ethylenimine units on OEI) = (5/5)/[(41.87-2)/4] ≈ 0.10, i.e. 10%.
